# THE ELDER MISTREATMENT EMERGENCY DEPARTMENT TOOLKIT: ADDRESSING ELDER MISTREATMENT DURING COVID-19 AND BEYOND

**DOI:** 10.1093/geroni/igac059.1592

**Published:** 2022-12-20

**Authors:** Rebecca Stoeckle, Kristin Lees Haggerty, Terry Fulmer

## Abstract

Elder Mistreatment (EM) is a prevalent problem that too often goes undetected. Hospital emergency departments (ED) offer a unique opportunity to screen for elder mistreatment and connect patients at risk with needed services but most do not have training and protocols in place to help staff screen for and respond to EM. In 2020, the National Collaboratory to Address Elder Mistreatment partnered with five hospitals to test the feasibility of implementing a streamlined set of tools and training known as the Elder Mistreatment Emergency Department Toolkit, and conducted two qualitative studies to better understand related barriers and facilitators to addressing EM. The presentations in this symposium share results from case studies at two feasibility test sites: Heywood Hospital, a small community hospital in central Massachusetts, and Lyndon B. Johnson Hospital, the busiest Level III Trauma Center in Texas, each presented by the respective site’s local clinical champion. Following presentations by the clinical champions, National Collaboratory expert core faculty will report results from two related qualitative studies. The first explores barriers and facilitators to communication between EDs and Adult Protective Services (APS) and the second explores older adult survivors’ own perspectives on the screening and response tools used in the feasibility study. The results of these studies highlight the need for streamlined and easy-to-use tools for identifying and responding to EM in busy EDs, the need for a personalized and trauma-informed approach to screening, and the importance of a personalized approach to connecting the ED and APS.

